# Genetic identification of SNP markers and candidate genes associated with sugarcane smut resistance using BSR-Seq

**DOI:** 10.3389/fpls.2022.1035266

**Published:** 2022-10-13

**Authors:** Qibin Wu, Yachun Su, Yong-Bao Pan, Fu Xu, Wenhui Zou, Beibei Que, Peixia Lin, Tingting Sun, Michael P. Grisham, Liping Xu, Youxiong Que

**Affiliations:** ^1^ Key Laboratory of Sugarcane Biology and Genetic Breeding, Ministry of Agriculture and Rural Affairs, National Engineering Research Center for Sugarcane, College of Agriculture, Fujian Agriculture and Forestry University, Fuzhou, China; ^2^ USDA-ARS, Southeast Area, Sugarcane Research Unit, Houma, LA, United States; ^3^ International College, Fujian Agriculture and Forestry University, Fuzhou, China

**Keywords:** sugarcane, smut resistance, BSR-seq, SNPs, key genes, expression pattern, molecular mechanism

## Abstract

Sugarcane smut caused by *Sporisorium scitamineum* is one of the most severe fungal diseases worldwide. In this study, a cross was made between a smut-resistant variety YT93-159 and a smut-susceptible variety ROC22, and 312 progenies were obtained. Two bulks of progenies were then constructed, one consisted of 27 highly smut resistant progenies and the other 24 smut susceptible progenies. Total RNAs of the progenies of each bulk, were pooled and subject to bulked segregant RNA-sequence analysis (BSR-Seq). A total of 164.44 Gb clean data containing 2,341,449 SNPs and 64,999 genes were obtained, 7,295 of which were differentially expressed genes (DEGs). These DEGs were mainly enriched in stress-related metabolic pathways, including carbon metabolism, phenylalanine metabolism, plant hormone signal transduction, glutathione metabolism, and plant-pathogen interactions. Besides, 45,946 high-quality, credible SNPs, a 1.27 Mb region at *Saccharum spontaneum* chromosome Chr5B (68,904,827 to 70,172,982), and 129 candidate genes were identified to be associated with smut resistance. Among them, twenty-four genes, either encoding key enzymes involved in signaling pathways or being transcription factors, were found to be very closely associated with stress resistance. RT-qPCR analysis demonstrated that they played a positive role in smut resistance. Finally, a potential molecular mechanism of sugarcane and *S. scitamineum* interaction is depicted that activations of MAPK cascade signaling, ROS signaling, Ca^2+^ signaling, and PAL metabolic pathway and initiation of the glyoxalase system jointly promote the resistance to *S. scitamineum* in sugarcane. This study provides potential SNP markers and candidate gene resources for smut resistance breeding in sugarcane.

## Introduction

Sugarcane (*Saccharum* spp. hybrids) is the largest cash sugar crop in the world with a global production of about 1.9 billion tons (Rajput et al., 2021) . It accounts for about 80% of the total sugar in the world and more than 90% in China with an important economic value (Lam et al., 2019; Rajput et al., 2021). Adversities such as drought, low temperature, high salinity, and diseases can seriously affect the yield and quality of sugarcane. Sugarcane smut caused by *Sporisorium scitamineum* is one of the most severe fungal disease worldwide (Padmanaban et al., 1988; Que et al., 2014a; Lam et al., 2019). The pathogenic mycelium of sugarcane smut invades the apical meristem through buds and produces a “black spike whip” at the tip of the plant, which is the most typical phenotypic trait. When the membrane is broken, numerous thick wall spores became available for disease re-infestation and spread (Nisha et al., 2004; Izadi and Moosawi-Jorf, 2007; Rajput et al., 2021). The severity of sugarcane smut is influenced by pathogenic microspecies, environmental conditions and cultivar characteristics (Akalach and Touil, 1996), and it becomes more severe under favorable conditions and in extreme cases even leading to a complete crop failure (Bachchhav et al., 1979). In addition to direct yield losses, sugarcane smut also causes significant reductions in sucrose content, purity, and other quality indicators (Kumar et al., 1989). The selection of smut-resistant varieties as parental crosses produces a high percentage of smut-resistant individuals in the progeny, so cultivation of smut-resistant varieties is a reliable and practicable measure to prevent and control this disease (Que et al., 2014b; Bhuiyan et al., 2021). However, sugarcane is an allopolyploid crop with a complex genetic background, and resistance to smut is likely to be determined by the cumulative effect of multiple master genes, numerous micro-effect genes and the interaction between sugarcane and *S. scitamineum* (Wu et al., 2013; Huang et al., 2018; Bhuiyan et al., 2021; Rajput et al., 2021; Ling et al., 2022). Therefore, to establish a rapid and efficient smut resistance breeding technology system, it is necessary to screen and identify as many resistance-linked molecular markers and key genes with potential breeding application value as possible.

In 1991, Michelmore et al. (1991) first proposed bulked segregant analysis (BSA) and successfully used it to screen for markers linked to target genes in *Lactuca sativa*. It is an efficient method for identifying markers closely linked to phenotypically related genes (Giovannoni et al., 1991). BSA combined with whole genome sequencing (BSA-Seq) was first used in model plants with small genomes such as *Arabidopsis thaliana* (Austin et al., 2011; Greenberg et al., 2011; Schneeberger and Weigel, 2011; Lindner et al., 2012;) and *Oryza sativa* (Abe et al., 2012; Takagi et al., 2013). In sugarcane, Wang et al. (2021) established a poly BSA-Seq approach using single-dose polymorphic markers, based on optimizing the traditional BSA-Seq, and further using this method, four molecular markers tightly linked to leaf blight (*Stagonospora tainanensis*) resistance were obtained, and 12 differentially expressed genes (DEGs) associated with leaf blight resistance were screened within a 1.0 Mb region of these molecular markers. However, BSA-Seq was costly for a complex large genomic species, especially the allopolyploid sugarcane. In 2012, Liu et al. (2012) reported a bulked segregation RNA-Seq (BSR-Seq) method, and successfully localized and cloned an epidermal wax synthesis gene *glossy3* in *Zea mays*. Currently, BSR-Seq has been widely used in the localization and cloning of genes in plants. Li et al. (2013) identified another epidermal wax synthesis gene *glossy13 via* BSR-Seq along with Seq-Walking. In *Triticum aestivum*, Zhu et al. (2020) constructed a mixed bulk of wheat resistant/susceptible powdery mildew (*Blumeria graminis* f. sp. *tritici*, *Bgt*) and successfully screened 3,816 differential single nucleotide polymorphisms (SNPs) and 3,803 DEGs by BSR-Seq, among which, 14 genes were up-regulated in the plant-pathogen interaction pathway. Ramirez-Gonzalez et al. (2015) constructed a mixed bulk with resistance/susceptibility to wheat yellow rust (*Puccinia striiformis* f. sp. *tritici*, *PST*) in polyploid wheat F_2_ generation and mapped the rust resistance gene *Yr15* to a 0.77-cM interval. In addition, the wheat stripe rust resistance genes *YrZH22* (Wang et al., 2017), *YrZM103* (Zhang et al., 2017), *Yr26* (Wu et al., 2018b), *Yr041133* (Li et al., 2022) were successfully localized and identified by RNA-Seq. In sugarcane, only Gao et al. (2022) constructed a genetic map of sugarcane-*S. scitamineum* interactions with an average distance of 1.96 cM using specific locus amplified fragment sequencing (SLAF-Seq) and BSR-Seq. The map contained 21 major QTLs with phenotypic variance explanation (PVE) of more than 8.0%, among which 10 QTLs were stable (repeatable) with PVEs ranging from 8.0 to 81.7%, and 77 SNPs from major QTLs were then converted to kompetitive allele specific PCR (KASP) markers, of which five were highly significantly linked to smut resistance in sugarcane.

In the present study, 312 F_1_ progenies were firstly created by crossing YT93-159 (smut-resistant, female) with ROC22 (smut-susceptible, male). After disease evaluation in field, 27 highly resistant and 24 susceptible progenies (5 highly susceptible and 19 susceptible) were selected to construct a resistant bulk and a susceptible bulk, respectively. Secondly, BSR-Seq analysis was used to obtain SNPs and DEGs. Thirdly, two algorithms, ΔSNP-index association analysis and Euclidean distance (ED), were used to identify and locate SNPs and smut resistance-related candidate genes. Fourthly, the expression patterns of candidate genes were analyzed to identify key genes associated with smut resistance by exploring the BSR-Seq data, the RNA-Seq data from different tissues of ROC22 (unpublished), the RNA-Seq data from YT93-159 and ROC22 infected with *S. scitamineum* for WGCNA analysis (Wu et al., 2022), and the real-time quantitative PCR (RT-qPCR) data. Finally, the BSR-Seq and WGCNA data were combined to depict the potential molecular mechanism of sugarcane and *S. scitamineum* interaction. This study is expected to set up a theoretical basis for exploring the molecular mechanism of sugarcane resistance to *S. scitamineum*, and to provide potential marker/gene resources for the molecular breeding of smut resistance in sugarcane.

## Materials and methods

### Smut disease evaluation of the F_1_ population

Mixed spores of sugarcane smut were collected from the Sugarcane Base in Baise, Guangxi (longitude 106°53’-107°26’E, latitude 23°16’-24°01’N) and the Experiment Station of Fujian Agriculture and Forestry University (FAFU), Fuzhou, Fujian (longitude 119°23’E, latitude 26°11’N), then stored in a 4°C refrigerator after drying.

A population of 312 F_1_ progenies was made from a cross between YT93-159 (smut resistant) and ROC22 (smut susceptible) in 2014. In February 2019, 60 double-bud stems of each progeny were packed in net bags, immersed in a smut spore suspension at a concentration of 5×10^6^ spores mL^-1^ for 15 min, removed and moistened at 25 to 28°C for 24 h. Then, 20 stems of each progeny were planted in a single row (3.0 m row length and 1.0 m row spacing) field plot in Baise and Fuzhou with three replications (Cang et al., 2021). During the growing season of 2019, the number of total and diseased plants within each plot were counted during field surveys and disease incidence rate was calculated (**Supplementary Table S2**). Level of resistance to sugarcane smut was classified according to percent of diseased plants (Que et al., 2006): highly resistant (0 to 3.0%), resistant 1 (3.1 to 6.0%), resistant 2 (6.1 to 9.0%), moderately resistant (9.1 to 12.0%), moderately susceptible (12.1 to 25.0%), susceptible 1 (25.1 to 35.0%), susceptible 2 (35.1 to 50.0%), highly susceptible 1 (50.1 to 75.0%), and highly susceptible 2 (75.1 to 100%).

### Pathogen stress treatment and bulks construction

**Figure 1:**
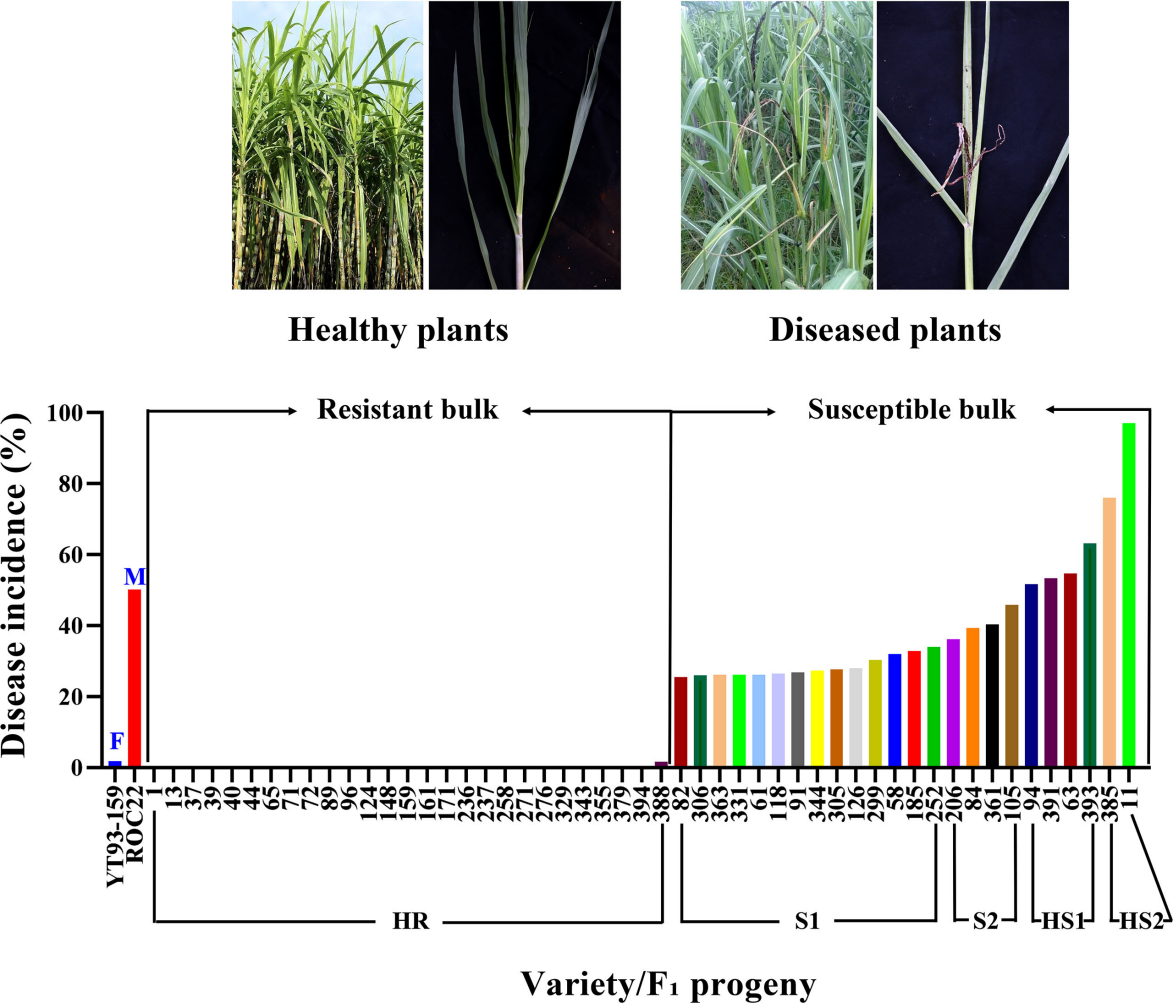
A graphic presentation of smut disease response by two selected extreme bulks of F_1_ progenies and two parental varieties (X-axis) based on disease incidence (%) (Y-axis). F, female parent; M, male parent; HR, highly resistance; S1, susceptible 1; S2, susceptible 2; HS1, highly susceptible 1; HS2, highly susceptible 2.

In December 2019, mature stems of 27 highly resistant progenies, 24 susceptible progenies (6 highly susceptible and 18 susceptible), and the two parental varieties were cut (**Figure 1** and **Supplementary Table S2**). In reference to Su et al. (2014), the stems were cut into single-bud setts, soaked in water for 24 h, and then germinated for 2-3 d in a greenhouse at 32°C/65% relative humidity (RH). When the shoots grew to 1-2 cm, they were syringe-inoculated with a 0.5 μL *S. scitamineum* spore suspension containing 5×10^6^ mL^-1^ in 0.01% (v/v) Tween-20. The inoculated shoots were cultured at 28°C/65% RH under alternative 12 h light/12 h darkness. At least six shoots were excised with three replications at 48 h post-infection, quickly frozen in liquid nitrogen, and stored at -80°C until total RNA extraction.

Total RNA was extracted from 53 samples (two parents, 27 highly resistant progenies and 24 susceptible progenies) using Trizol reagent (Invitrogen, CA, USA) following the manufacturer’s protocol. The total RNA of YT93-159 was designated as T01. The total RNA of ROC22 was designated as T02. Equal amounts of total RNA from the 27 highly resistant progenies were mixed to form the resistant bulk as T03. Equal amounts of total RNA from the 24 susceptible progenies were mixed to form the susceptible bulk as T04. After validation by agarose gel electrophoresis and NanoDrop One (Thermo Fisher Scientific, Waltham, MA, USA), the four total RNA samples were sent to Baimaike Biotechnology Co., Ltd. (Beijing, China) for cDNA library construction.

### Library construction and BSR sequencing

RNA concentration and integrity number (RIN) of the four total RNA samples were measured with an Agilent 2100 Bioanalyzer (Agilent Technologies, Palo Alto, CA, USA). The requirements for cDNA library construction and subsequent sequencing were met when the RIN was ≥7. mRNA was enriched with magnetic beads with Oligo (dT) and then randomly interrupted by the addition of fragmentation buffer. Double-stranded cDNAs were synthesized using TruSeq Stranded mRNA LT Sample Prep Kit (Illumina, CA, USA), purified, end-repaired, A-tailed, sequenced, fragment size selected, and finally enriched by PCR to obtain cDNA libraries (Bolger et al., 2014). Libraries were analyzed by Agilent Bioanalyzer 2100 and then sequenced by Illumina HiSeq™ (Beijing Baimaike Biotechnology Co., Ltd., China). Sequencing depth was set at an average of 15 GB of clean data per parent and 60 GB of clean data per bulk.

Quality of sequencing data was assessed using FastQC and Trimmomatic software (Kim et al., 2015). Raw data were filtered to remove adapter reads, low quality reads, joint sequences, and ribosomal RNAs to obtain high quality clean reads (Bolger et al., 2014). The whole genome sequence of *Saccharum spontaneum* (sspon_v201901030) (Zhang et al., 2018) was used as the reference genome. The STAR (v2.3.0e) software (Dobin et al., 2013) was used to align the clean reads with the reference genome to obtain mapped reads for subsequent analysis.

### Gene structure optimization, alternative splicing analysis, and discovery of new genes

Due to data and software limitations, the annotation of reference genomes is often not sufficiently accurate, and the original annotated gene structure needs to be optimized. Based on the annotation information of the *S. spontaneum* genome, the untranslated region (UTR) was extended upstream and downstream to correct the gene boundary if the region outside the original gene boundary was supported by continuous mapped reads. The Cufflinks v2.2.1 software (Florea et al., 2013) was used to splicing the alignment results, and compared with the initial annotation results to discover new transcripts or new genes that were not originally annotated. Then, the ASprofile v1.0.4 software (Trapnell et al., 2010) was used to obtain types and corresponding numbers of alternative splices. The BLAST software (Ye et al., 2006) was also applied to annotate the new transcripts or new genes with functions from several databases such as NR (RefSeq non-redundant proteins) (Deng et al., 2006), SWISS-PROT (http://www.expasy.org/sprot/ and http://www.ebi.ac.uk/swissprot/) (Boeckmann et al., 2003), GO (Gene Ontology) (Ashburner et al., 2000), KEGG (Kyoto Encyclopedia of Genes and Genomes) (Kanehisa et al., 2004), COG (Clusters of Orthologous Groups) (Tatusov et al., 2000), KOG (Clusters of orthologous groups for eukaryotic complete genomes) (Tatusov et al., 2000), eggNOG (evolutionary genealogy of genes: Non-supervised Orthologous Groups) (Jensen et al., 2007) and Pfam 35.0 (http://pfam.xfam.org/) (Mistry et al., 2021).

### Analysis and annotation of differentially expressed genes

FPKM (fragments per KB of transcript per million fragments mapped) was used as a measure of transcription or gene expression level (Garber et al., 2011) after the clean reads were aligned to *S. spontaneum* genome sequence to obtain the corresponding positional information. EBSeq v1.6.0 (Leng et al., 2013) was used to obtain DEGs between the two samples when fold change was > 2 and false discovery rate (FDR) was< 0.01. Statistical and clustering analyses were performed on DEGs among the parents and the two bulks to present genome-wide expression patterns including candidate regions (Zhu et al., 2020), and functional DEGs annotation.

### SNP detection and mining of candidate regions associated with smut resistance

SNPs were detected following the reference flowchart of GATK v3.2-2 software (McKenna et al., 2010). Briefly, based on the localization of clean reads along with the *S. spontaneum* genome, GATK was used to perform local realignments and base recalibrations to ensure the accuracy of the SNPs. Then, SNPs with multiple genotypes,< four support degree, consistency between the resistant and the susceptible bulks, and inconsistency between the parents and corresponding bulks were filtered to obtain high quality and credible SNPs (Reumers et al., 2012).

SNP-index is a method used mainly to find significant genotype frequency differences between mixed bulks (Trapnell et al., 2010). The difference is counted by the ΔSNP-index. The closer the ΔSNP-index is to 1, the stronger the association of the SNP marker with the trait. To eliminate false positive loci, the ΔSNP-index was fitted using the SNPNUM method (Altschul et al., 1997) based on the SNPs’ positions on the reference genome. Then, the ΔSNP-index between resistant and susceptible parents and the bulks was calculated for each SNP using the following formula:


ΔSNP−index = (SNP–index of resistant parent/resistant bulk) − (SNP–index of susceptible parent/susceptible bulk)

Referring to the method of Takagi et al. (2013), the threshold for SNP detection was set as a test of 100,000 permutations in coupling with a 99% confidence. SNPs with larger than the threshold ΔSNP-index values (set as 0.05 in this study) in candidate regions were selected as candidate loci that were associated with smut resistance in sugarcane.

The Euclidean distance (ED) is a method that uses RNA-Seq data to find markers significantly different between mixed bulks and to assess regions of association with traits (Trapnell et al., 2010). To eliminate background noise, the original ED value was processed to (ED)^2^ (Altschul et al., 1997), which was taken as the correlation value. The median +3 × standard deviation (set as 0.04 in this study) of the fitted values for all loci was taken as the correlation threshold for analysis (Trapnell et al., 2010). In this study, ΔSNP-index and ED analysis were used to screen for genes and candidate regions associated with sugarcane smut resistance. The common non-synonymous mutant genes obtained by both methods were removed, and all the remaining non-synonymous mutant genes were identified as candidate genes.

### Functional annotation and expression analysis of candidate genes

The conserved structural domains of the candidate genes from the candidate regions were analyzed using the NCBI database (Marchler-Bauer et al., 2017). The functions of the candidate genes were annotated according to *Arabidopsis* homologs from the TAIR database (https://www.arabidopsis.org/). To identify the key genes, the expression patterns of the candidate genes were analyzed by the BSR-Seq data, RNA-Seq data of five different tissues (root, epidermis, pith, leaf, and bud) of ROC22 (unpublished), and the WGCNA data of YT93-159 and ROC22 infected by *S. scitamineum* (Wu et al., 2022), and the expression heat map of the key genes was plotted using TBtools (Chen et al., 2020).

### Validation of key genes by RT-qPCR

On 0 d, 1 d, 2 d, and 5 d post *S. scitamineum* inoculation, the shoots of YT93-159 and ROC22 were sampled as described earlier for RT-qPCR analysis. Twenty primer pairs were designed for the key genes by Beacon Designer 8.0 (**Supplementary Table S1**). *GAPDH* was used as the internal reference gene (Iskandar et al., 2004). At each time point, three independent biological replicates were taken. RT-qPCR reactions was performed on ABI QuantStudio™ 3 system (Thermo Fisher Scientific, Waltham, MA, USA). Total reaction volume was 25 µL, containing 12.5 µL FastStart Universal SYBR Green PCR Master (Roche, Shanghai, China), 0.5 µL of each primer (10 µM), 1.0 µL template (10×cDNA diluted liquid), and 10.5 µL ddH_2_O. The thermal cycling program was: 50°C for 2 min; 95°C for 10 min; and 40 cycles of (95°C for 15 s; 60°C for 1 min). Expression levels of the key genes were calculated using the 2^−ΔΔCT^ method (Livak and Schmittgen, 2001). Histograms were graphed by GraphPad Prism 6. Significance (*p* < 0.05) and standard error (SE) were determined by the Duncan’s new multiple range test.

## Results

### Field survey of smut disease

Smut disease survey data were shown in **Supplementary Table S2**. The disease grade of each F_1_ progeny was assigned according to Que et al. (2006). Based on the grades, 27 progenies were selected to constitute the resistant bulk and 24 progenies (6 highly susceptible and 18 susceptible) were selected to form the susceptible bulk (**Figure 1** and **Supplementary Table S2**).

### BSR-Seq analysis

A total of 164.44 GB clean data were obtained from the four samples by BSR-seq analysis, including 17.46 GB from YT93-159 (T01), 18.49 GB from ROC22 (T02), 60.19 GB from the resistant bulk (T03), and 68.28 GB from the susceptible bulk (T04). The GC content ranged from 52.20% to 54.02% and the Q30 base percentage was above 93.27% (**Table 1**). The efficiency of sequence alignment to the *S. spontaneum* reference genome was 74.34% for T01, 74.05% for T02, 73.57% for T03, and 71.40% for T04, respectively (**Table 1** and **Supplementary Figure S1**). These results indicated that the quality of the sequencing data was high and met with the requirements of subsequent analysis.

**Table 1:** BSR-seq data summary for YT-93-159 (T01), ROC22 (T02), the resistant bulk (T03), and the susceptible bulk (T04).

Item	T01	T02	T03	T04
Clean reads	58,396,553	61,866,714	201,100,419	228,225,596
Clean bases	17,460,639,520	18,499,059,990	60,196,617,960	68,285,233,824
Q30 (%)	93.61	93.62	94.04	93.27
GC (%)	52.20	54.02	52.92	53.62
Total reads	116,793,106	123,733,428	402,200,838	456,451,192
Mapped reads	86,818,606 (74.34%)	91,620,775 (74.05%)	295,900,029 (73.57%)	325,907,122 (71.40%)

### Gene structure optimization, alternative splicing analysis, and discovery of new genes

**Figure 2:**
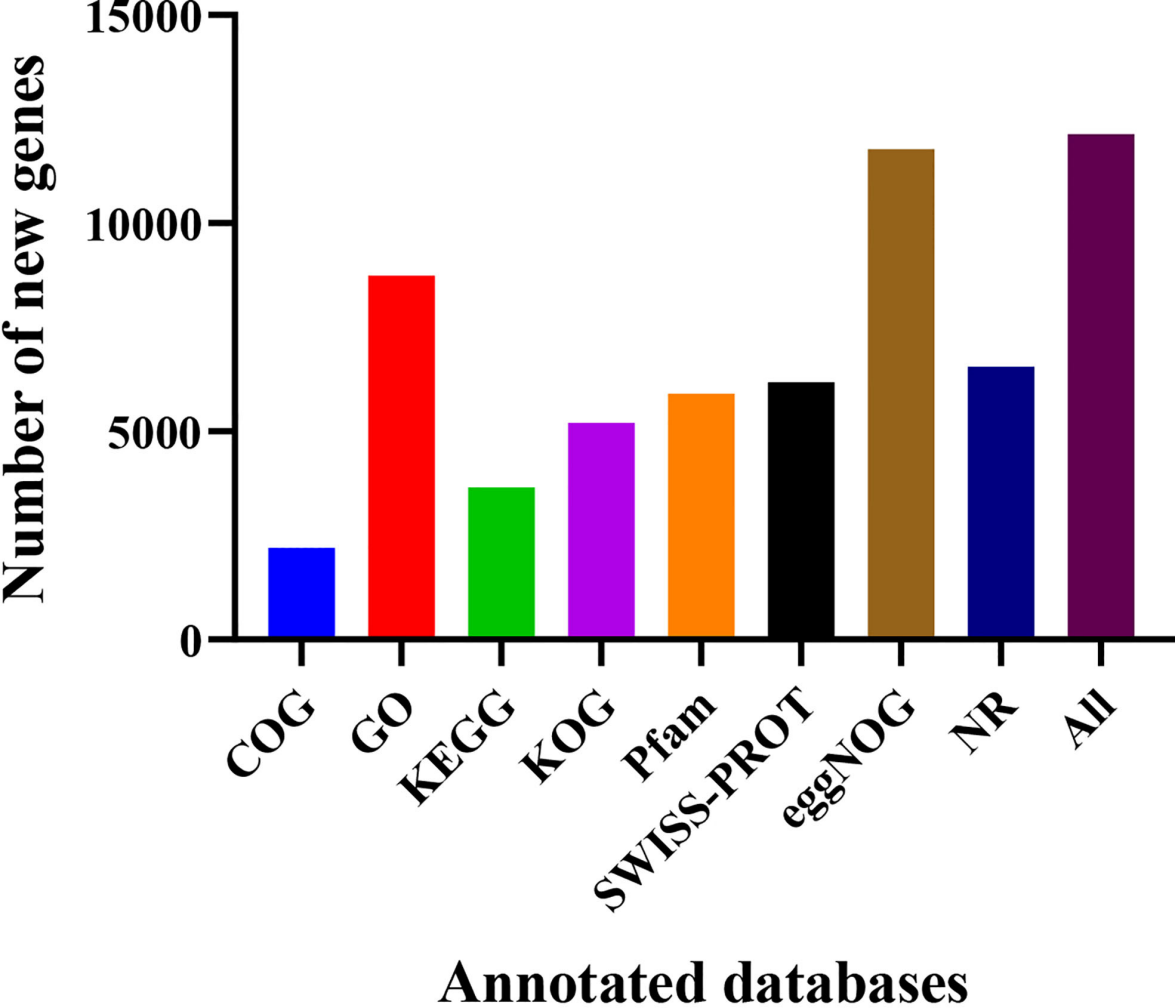
Number of new genes functionally annotated in various databases.

Gene locations and size comparisons were conducted in reference to the *S. spontaneum* reference genome. A total of 17,477 genes were optimized at the 5’ and 3’ untranslated regions (UTRs) (**Supplementary Table S3**). Alternative splicing types were divided into seven categories by the ASprofile software (Trapnell et al., 2010), namely, transcription start site (TSS), transcription terminal site (TTS), single exon skipping (SES), multi exon skipping (MES), single intron retention (SIR), multi-intron retention (MIR), and alternative exon ends (AE). As shown in **Table 2**, 170,932, 170,452, 176,098, and 176,722 alternative splicing transcripts were obtained for YT93-159 (T01), ROC22 (T02), the resistant bulk (T03), and the susceptible bulk (T04), respectively. TSS accounted for the most, followed by TTS. TSS and TTS together accounted for about 90% of all alternative splicing. A total of 17,066 new genes were identified after filtering out sequences encoding peptide chains that were too short (less than 50 amino acid) or containing only one exon. Finally, 12,138 new genes were annotated upon blasting with various databases (**Figure 2**).

**Table 2:** Number and type of alternative splicing.

**Number/Sample**	**T01**	**T02**	**T03**	**T04**
TSS	79,195 (46.33%)	79,032 (46.37%)	81,159 (46.09%)	81,328 (46.02%)
TTS	76,526 (44.77%)	76,610 (44.95%)	77,262 (43.87%)	77,629 (43.93%)
SES	4,465 (2.61%)	4,454 (2.61%)	5,102 (2.90%)	5,343 (3.02%)
MES	1,199 (0.70%)	1,222 (0.72%)	1,410 (0.80%)	1,384 (0.78%)
SIR	2,974 (1.74%)	2,670 (1.57%)	3,484 (1.98%)	3,478 (1.97%)
MIR	191 (0.11%)	194 (0.11%)	271 (0.15%)	272 (0.15%)
AE	6,382 (3.73%)	6,270 (3.68%)	7,410 (4.21%)	7,288 (4.12%)
SUM	170,932	170,452	176,098	176,722

### Analysis and annotation of differentially expressed genes

**Figure 3:**
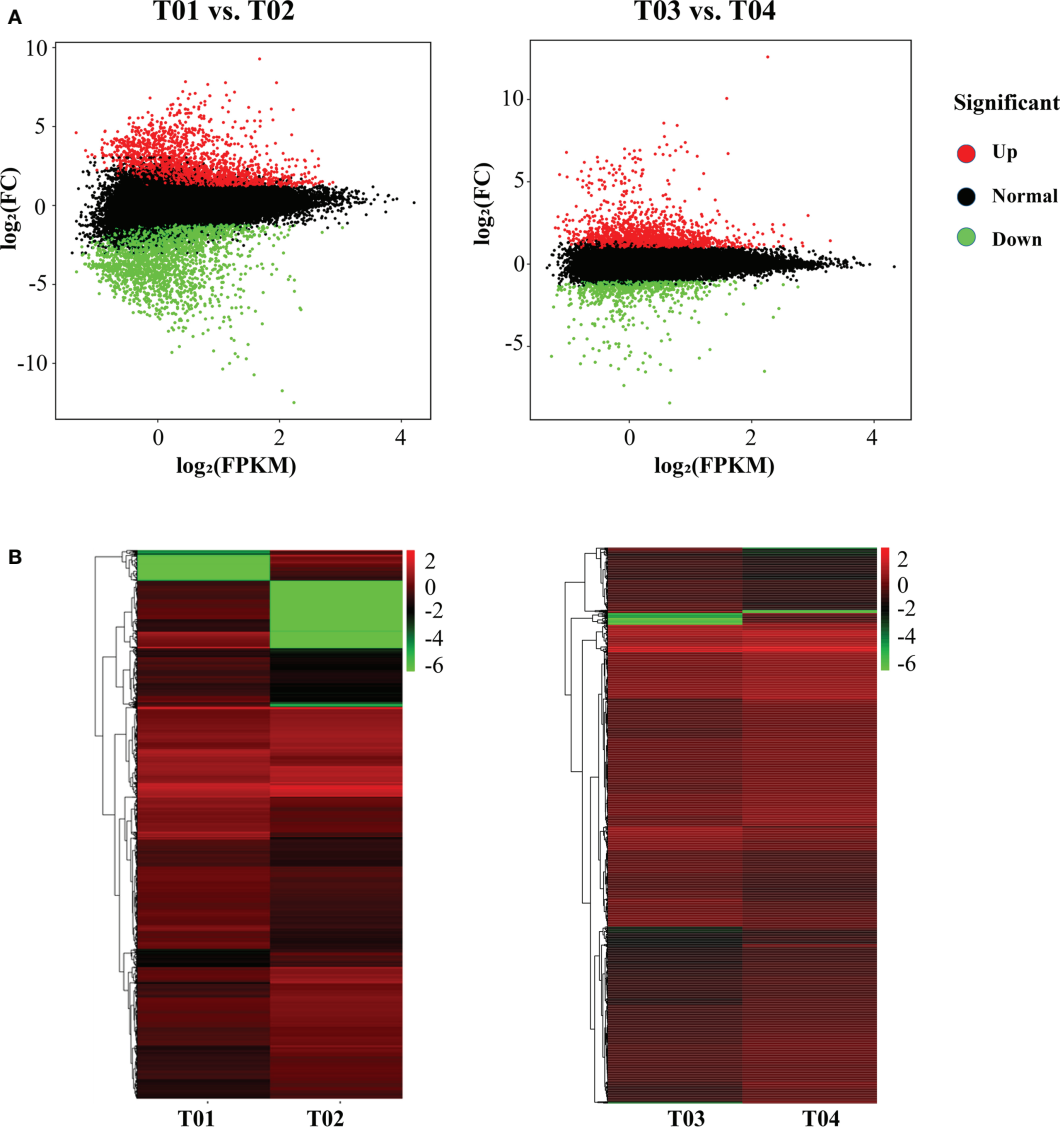
MA plot **(A)** and cluster plot **(B)** of differentially expressed genes (DEGs) between YT93-159 (T01) and ROC22 (T02) and between the resistant bulk (T03) and the susceptible bulk (T04).

**Figure 4:**
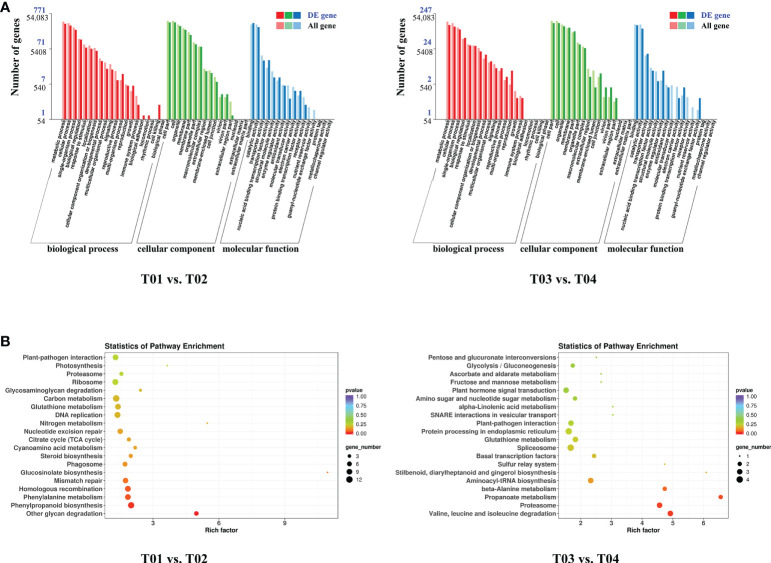
GO **(A)** and KEGG **(B)** enrichment analyses of DEGs between YT93-159 (T01) and ROC22 (T02) and between the resistant bulk (T03) and the susceptible bulk (T04).

A total of 64,999 genes were identified by BSR-Seq sequencing, of which 7,295 were DEGs (**Figure 3**). The number of DEGs between the two parents (T01 vs. T02) was 5,166, of which 2,350 were up-regulated and 2,816 were down-regulated (**Figure 3B**). The number of DEGs between the resistant and the susceptible bulks (T03 vs. T04) was 2,636, of which 1,816 were up-regulated and 820 were down-regulated (**Figure 3B**). GO enrichment analysis showed that only 1,018 DEGs could be significantly enriched (**Figure 4A**). The 771 DEGs between the two parents (T01 vs. T02) (**Supplementary Table S4**) and the 247 DEGs between the resistant bulk and the susceptible bulk (T03 vs. T04) (**Supplementary Table S5**) were mainly enriched in metabolic process, cellular process, single-organism process of the biological process (BP) category, cell, cell part, organelle of the cellular component (CC) category, binding, catalytic activity, and transporter activity of the molecular function (MF) category (**Figure 4A**).

KEGG enrichment analysis showed that DEGs between the two parents (T01 vs. T02) and between the resistant bulk and the susceptible bulk (T03 vs. T04) were enriched in 90 and 40 metabolic pathways, respectively (**Figure 4B**). The DEGs between the two parents (T01 vs. T02) were mainly enriched in ribosome, plant-pathogen interaction, phenylpropanoid biosynthesis, phenylalanine metabolism, homologous recombination, glutathione metabolism, DNA replication, and carbon metabolism (**Figure 4B** and **Supplementary Table S6**). The DEGs between the resistant bulk and the susceptible bulk (T03 vs. T04) were mainly enriched in spliceosome, protein processing in endoplasmic reticulum, proteasome, plant-pathogen interaction, plant hormone signal transduction, glutathione metabolism, and aminoacyl-tRNA biosynthesis (**Figure 4B** and **Supplementary Table S7**). Overall, the DEGs were mainly enriched in stress-related metabolic pathways, such as carbon metabolism, phenylpropanoid biosynthesis, phenylalanine metabolism, plant hormone signal transduction, glutathione metabolism, and plant-pathogen interactions.

### Mining SNPs and candidate regions associated with smut resistance

**Figure 5:**
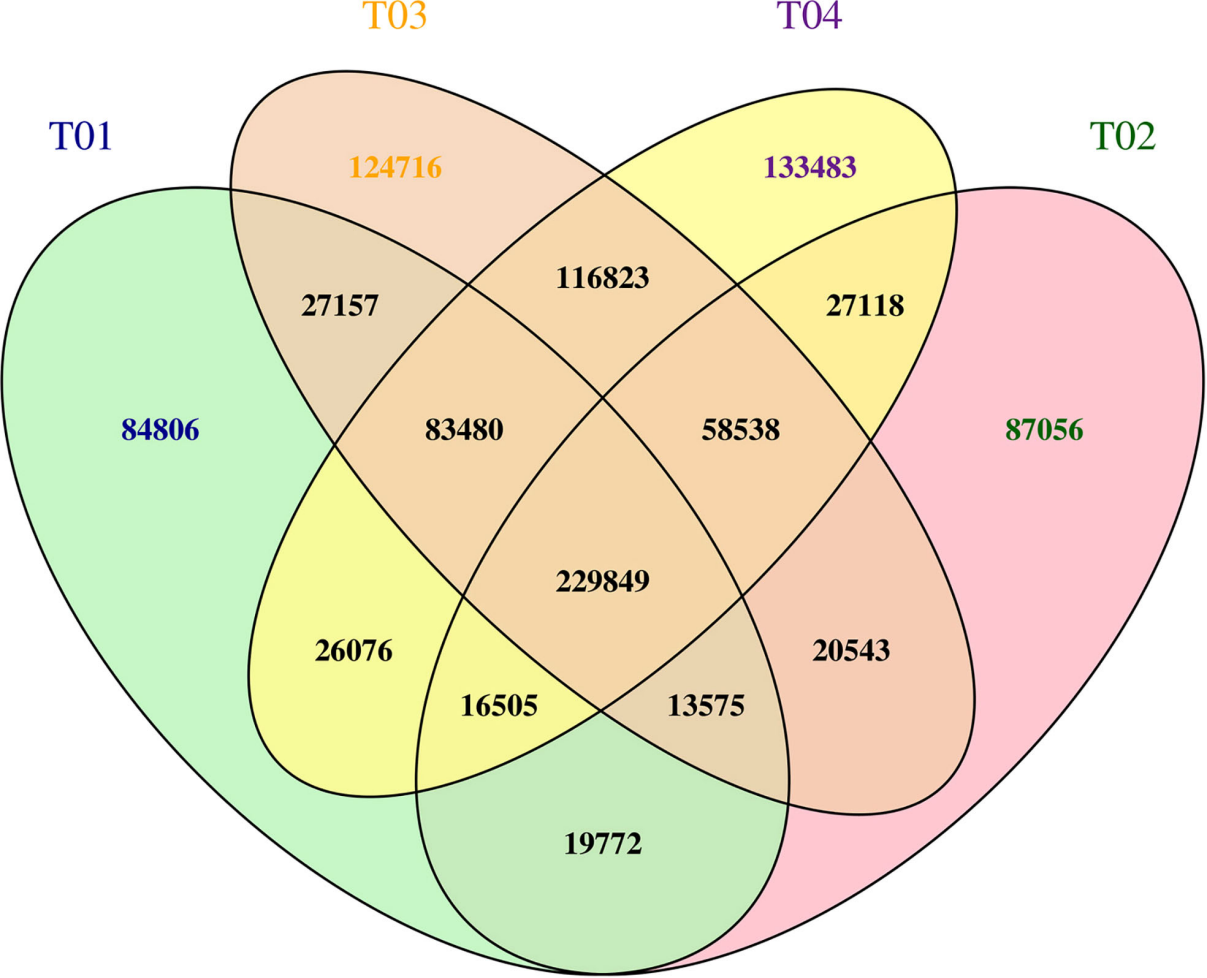
** **A Venn plot of SNP statistics for T01, T02, T03, and T04. The numbers of SNPs are shown.

Mining SNPs from the BSR-Seq data resulted in a total of 2,341,449 SNPs, including 501,220 from YT93-159 (T01), 472,956 from ROC22 (T02), 674,681 from the resistant bulk (T03), and 692,232 from the susceptible bulk (T04) (**Figure 5**). After filtering out the SNPs common to all four samples, a total of 1,069,497 SNPs was remained. Then SNPs with multiple genotypes (2,689), with support degree< 4 (505,477), with consistent genotypes between the resistant bulk and the susceptible bulks (158,595), with inconsistency between the two parents or between the two corresponding bulks (356,790) were filtered out, leaving 45,946 high quality, credible SNPs for ΔSNP-index and ED analyses (**Figure 5**).

**Figure 6:**
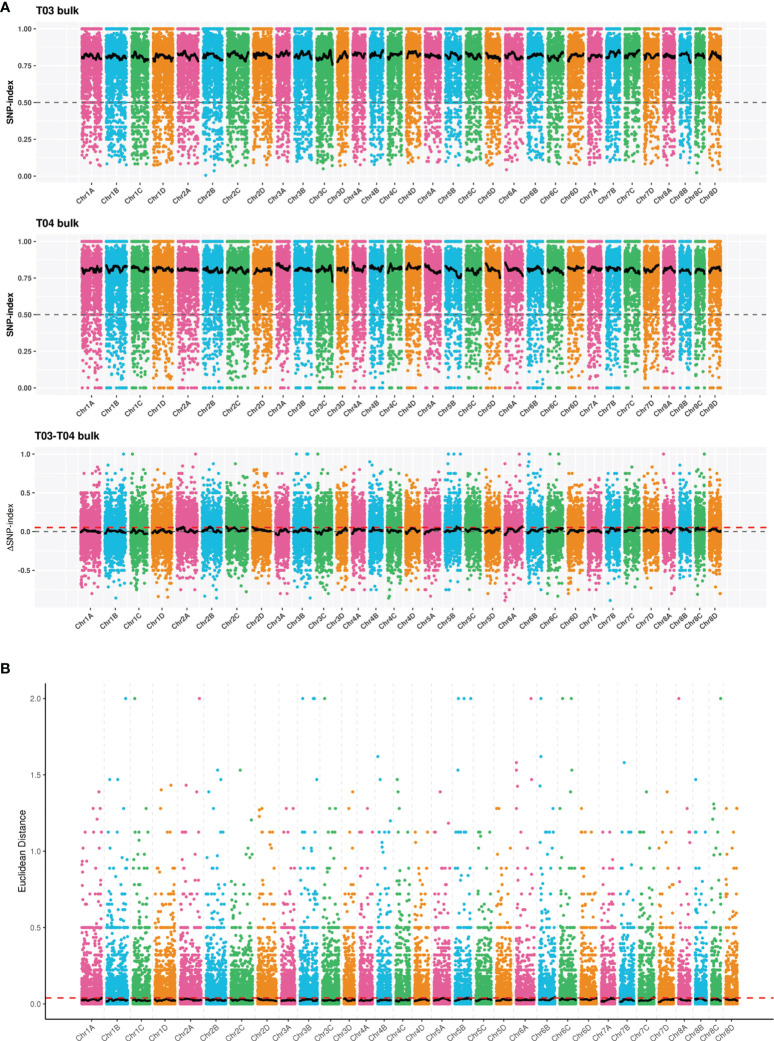
Distribution of correlation values for ΔSNP-index **(A)** and Euclidean distance (ED) **(B)** methods on the *S. spontaneum* chromosomes. The colored dots represent the ΔSNP-index or ED value, the black line represents the fitted ΔSNP-index or ED value, and the red dotted line represents the significance threshold.

A total of 32 candidate regions associated with smut resistance were identified by the ΔSNP-index method. The total length of these regions was 36.37 Mb containing 889 genes and 103 non-synonymous mutant genes (**Figure 6A** and **Supplementary Table S8**). On the other hand, the ED algorithm analysis identified 15 candidate regions associated with smut resistance with a total length of 10.40 Mb, 237 genes, and 30 non-synonymous mutant genes (**Figure 6B** and **Supplementary Table S9**). Combining the results from both ΔSNP-index and ED analyses resulted in a 1.27 Mb region that was localized between 68,904,827 and 70,172,982 on *S. spontaneum* chromosome Chr5B. This region contained 21 genes and 4 non-synonymous mutant genes. After removing the common non-synonymous mutant genes obtained by both ΔSNP-index and ED methods, all the remaining non-synonymous mutant genes were regarded as candidate genes. Ultimately, a total of 129 candidate genes associated with smut resistance were identified.

### Functional annotation of candidate genes

To obtain functional information, the conserved structural domains of 129 candidate genes were analyzed by Conserved Domain Database in NCBI (Marchler-Bauer et al., 2017) and the homologous functions of these genes in *Arabidopsis* were annotated with TAIR database (**Supplementary Table S10**). The results showed that among the 129 candidate genes, 24 either code key enzymes involved in signaling pathways or are transcription factors that closely relate to plant stress resistance. The examples include the PHD-type (*PHD-ZFP*) and BED-type zinc finger proteins (*BED-ZFP*), ethylene response factor (*ERF*), eukaryotic translation initiation factor 2b (*eIF2b*), *WRKY53*, and *MYB* transcription factors; *MAPK*, *MEK*, and *Raf-like* genes in mitogen-activated protease kinase signaling pathway; calmodulin 42 (*CML42*) and calcineurin B-like interacting protein kinase (*CIPK*) genes in calcium (Ca^2+^) signaling pathway; the glyoxalase I 10 (*GLYI10*) and glyoxalase II 21 (*GLYII21*) genes in the glyoxalase system; the peroxidase (*POD*) and catalase 1 (*CAT1*) genes in reactive oxygen species (ROS) signaling pathway; and other protease genes such as leucine-rich repeat receptor-like protein kinase (*LRR-RLK*), cyclin-dependent kinase (*CDK*), lipoxygenase (*LOX*), 4-coumarate:CoA ligase (*4CL*), serine protease inhibitor (*SPI*), purple acid phosphatase (*PAP*), glucose transporter (*GLUT*), potassium transporter (*KUP*), and ankyrin-like (*ANK*).

### Expression pattern analysis of key genes

**Figure 7:**
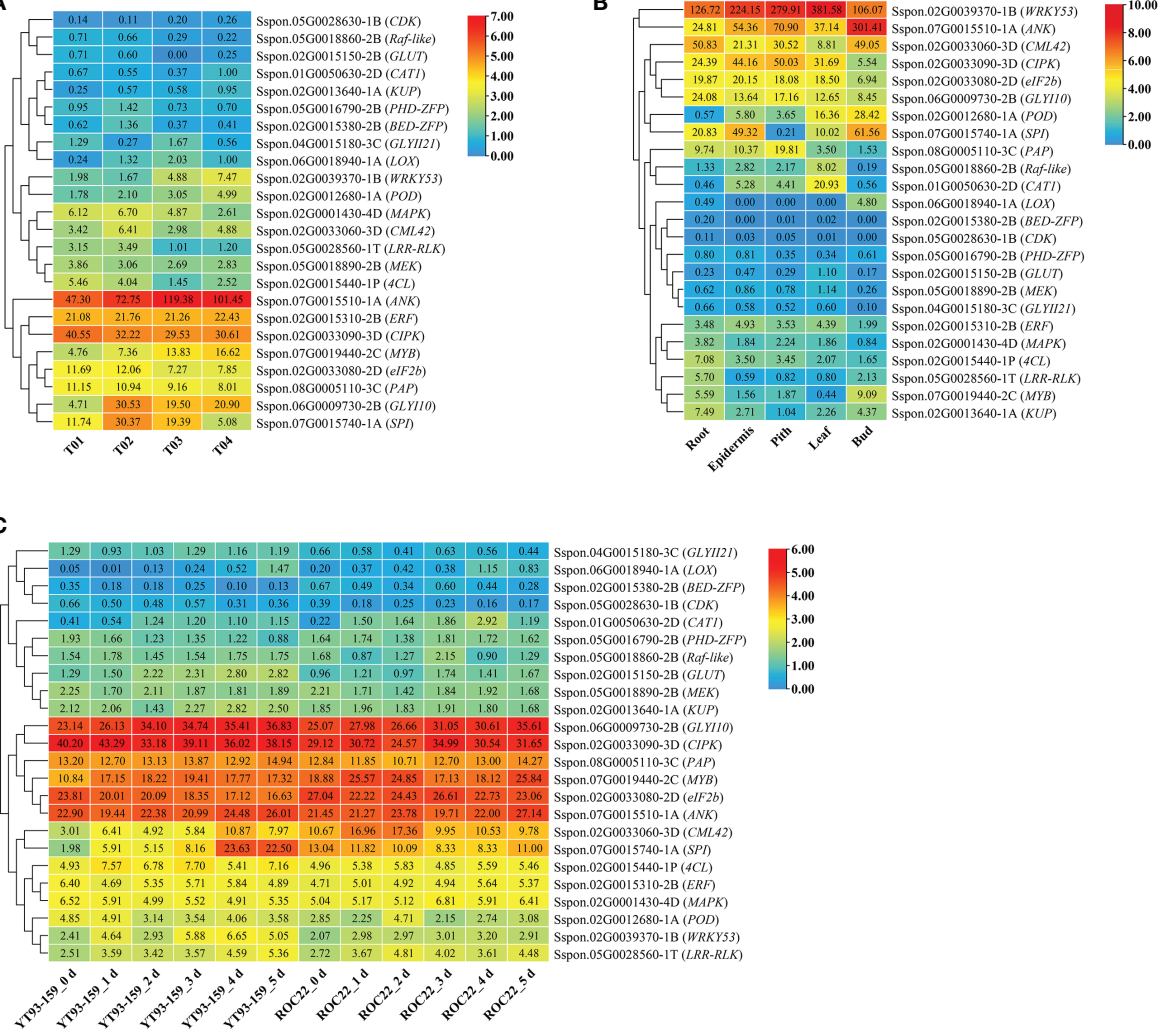
Expression patterns of key genes based on BSR-Seq database **(A)** in ROC22 **(B)**, and under the stress of sugarcane smut pathogen **(C)**.

As shown in **Figure 7A**, 24 key genes were expressed in all four samples, namely, YT93-159 (T01), ROC22 (T02), the resistant bulk (T03) and the susceptible bulk (T04). The expression levels of *ERF*, *MYB*, *eIF2b* transcription factors, and protease genes (*ANK*, *CIPK*, *PAP*, *SPI*, and *GLYI10*) were high, but low for *CDK*, *Raf-like*, *GULT*, *CAT1*, and *KUP* (**Figure 7A** and **Supplementary Table S11**). Twenty-one key genes were expressed in all the five tissues of ROC22, among which *WRKY53*, *ANK*, *CML42*, *CIPK*, *eIF2b*, and *GLYI10* expressed at a high level, while *BED-ZFP*, *CDK*, *PHD-ZFP*, *GLUT*, *MEK*, and *GLYII21* expressed at very low level (**Figure 7B**). *LOX* only expressed in buds. *MYB* mostly expressed in buds. *Raf-like* and *CAT1* mostly expressed in leaves. *4CL*, *LRR-RLK*, and *KUP* mostly expressed in roots **(** **Figure 7B**). The WGCNA data (Wu et al., 2022) collected at six time points post *S. scitamineum*-inoculation of YT93-159 and ROC22 showed that *GLYI10*, *CIPK*, *PAP*, *MYB*, *eIF2b*, and *ANK* expressed at high levels, followed by *CML42*, *SPI*, *4CL*, *ERF*, *MAPK*, *POD*, *WRKY53*, and *LRR-RLK*, while *GLYII21*, *LOX*, *BED-ZFP*, and *CDK* expressed at low levels (**Figure 7C**). Both *CIPK* and *WRKY53* expressed at higher levels in the smut-resistant variety than in the susceptible variety, while the opposite was true for *eIF2b* and *CML42*. In addition, the expression level of *GLYI10* increased gradually post *S. scitamineum*-inoculation and peaked at 5 d (**Figure 7C**).

### RT-qPCR validation of key genes

**Figure 8:**
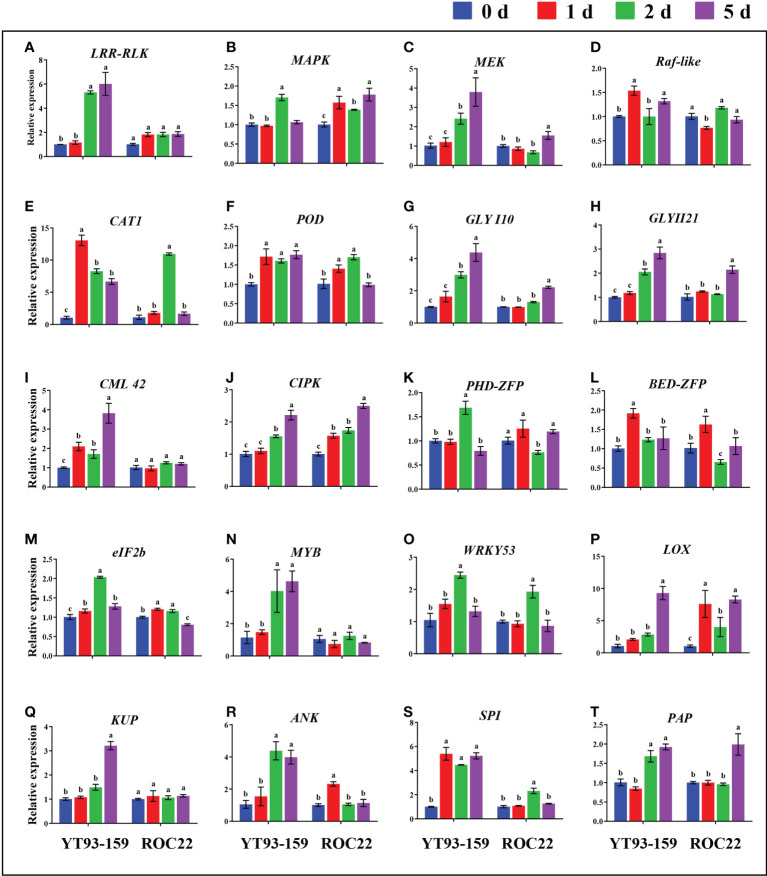
Comparative expression analysis of 20 key genes between YT93-159 and ROC22 at 0 d (blue), 1 d (red), 2 d (green), and 5 d (purple) post *S. scitamineum*-inoculation by RT-qPCR. Different letters indicate a significant difference at 5% level (*p* ≤ 0.05). All data points are mean ± standard error (*n* = 3). **(A)** LRR-RLK; **(B)** MAPK; **(C)** MEK; **(D)** Raf-like; **(E)** CAT1; **(F)** POD; **(G)** GLYI10; **(H)** GLYII21; **(I)** CML42; **(J)** CIPK; **(K)** PHD-ZFP; **(L)** BED-ZFP; **(M)** eIF2B; **(N)** MYB; **(O)** WRKY53; **(P)** LOX; **(Q)** KUP; **(R)** RNK; **(S)** SPI; **(T)** PAP.

Only 20 key genes were successfully amplified by RT-qPCR. All the 20 key genes could be induced by *S. scitamineum* infection. The expression levels of 15 genes were remarkably higher in YT93-159 than ROC22 (**Figure 8** and **Supplementary Table S12**). In YT93-159, the expression level of *LRR-RLK*, *MEK*, *CML42*, *MYB*, and *KUP* increased gradually, and peaked on 5 d at a rate of 6.01-, 3.80-, 3.82-, 4.63-, and 3.21-fold higher than the control (0 d), respectively. However, their expression levels were largely unchanged or slightly up-regulated in ROC22 (**Figures 8A, C, I, N, Q** **)**. Similarly, the expression level of *GLYI10* and *GLYII21* was higher in YT93-159 than in ROC22, gradually increased from 0 d to 5 d post *S. scitamineum*-inoculation and reached the peak at 5 d (**Figures 8G, H** **)**. The expressions of *Raf-like*, *CAT1*, *BED-ZFP*, and *SPI* were up-regulated upon *S. scitamineum* inoculation and reached the peak at 1 d post inoculation (**Figures 8D, E, L, S**), while the expressions of *PHD-ZFP*, *eIF2b*, *WRKY53*, and *ANK* reached the peak at 2 d post inoculation (**Figures 8K, M, O, R**). In addition, genes *MAPK*, *POD*, *CIPK*, *LOX*, and *PAP* were also induced to express upon *S. scitamineum* inoculation at similar levels among different samples (**Figures 8B, F, J, P, T**). These results indicated that the expression levels of all 20 key genes were up-regulated by varying degrees upon *S. scitamineum* inoculation and that the expression patterns were certainly different between sugarcane varieties either resistant or susceptible to the smut disease.

## Discussion

Sugarcane is an allopolyploid plant with a complex genetic background and an extremely low recombination rate of excellent genes. The resistance to sugarcane smut is likely to be determined by the cumulative effect of multiple master genes, numerous micro-effect genes, and the interaction between sugarcane and the smut pathogen *S. scitamineum* (Wu et al., 2013; Huang et al., 2018; Bhuiyan et al., 2021; Rajput et al., 2021; Ling et al., 2021). It is thus of great significance to mine and identify molecular markers and key genes in sugarcane genome that associate with smut resistance. At present, BSR-Seq has been widely used in localizing and cloning of genes in plants (Liu et al., 2012; Li et al., 2013; Zhu et al., 2020). We applied this technique in sugarcane and found 45,946 high quality and credible SNP markers, a 1.27 Mb chromosome region Chr5B, and 129 candidate key genes that associated with smut resistance (**Supplementary Table S10**). We also explored the NCBI and TAIR databases to functionally annotated 24 key genes, of which 20 were validated by RT-qPCR (**Figure 8**). These key genes play an important role in sugarcane’s response to *S. scitamineum* infection through the glyoxalase system, MAPK cascade signaling, ROS signaling, Ca^2+^ signaling, and other resistance-associated metabolic pathways.

The glyoxalase enzyme system, including GLYI and GLYII, is the most efficient way to remove excess toxic MG and is important to cope with various abiotic stresses and pathogen infections in plants (Tardieu and Tuberosa, 2010). Ghosh and Islam (2016) reported that the expression of *GmGLYI-6*, GmGLYI-9 *GmGLYI-20*, *GmGLYII-6*, and *GmGLYII-10* were up-regulated when soybean plants were infected by various pathogens. Similarly, Yan et al. (2018) showed that under *Plasmodiophora brassicae* infestation, *BrGLYI1*, *BrGLYI2*, *BrGLYI6*, *BrGLYI11*, *BrGLYI16*, *BrGLYII8*, and *BrGLYII10* genes of *Brassica rapa* were induced to express at an up-regulated level. Álvarez Viveros et al. (2013) found that simultaneous transfer of overexpressed *GLYI* and *GLYII* genes into *Solanum lycopersicum* Mill. reduced its oxidative stress response and thus improved its tolerance to salt stress. Singla-Pareek et al.(2003); Singla-Pareek et al.(2006) and Singla-Pareek et al.(2008) reported that simultaneous transfer of overexpressed *GLYI* and *GLYII* genes into *Nicotiana tabacum* helped improve its tolerance to heavy metals and salt stress. Our previous study also indicated that the sugarcane *SoGloI* gene played a role in sugarcane’s response to various biotic and abiotic stresses (Wu et al., 2018a). In this study, during 0 d to 5 d post *S. scitamineum*-infection in both varieties YT93-159 and ROC22, the expression level of *GLYI10* and *GLYII21* genes was continuously up-regulated by approximately 4.38-fold and 2.84-fold comparing to the control at 5 d, respectively. The expression level of *GLYI10* and *GLYII21* was also higher in resistant variety YT93-159 than susceptible variety ROC22 (**Figures 8G, H** **)**, confirming that the *GLYI10* and *GLYII21* genes are responding to *S. scitamineum* infection by scavenging excess toxic MG.

The MAPK cascade signaling pathway is prevalent and highly conserved in plants. It involves three protein kinases, MAPKKK, MAPKK, and MAPK, in functional tandem (Hwa and Yang, 2008). When plants were infected with various pathogens, PAMPs bind to RLKs to activate MPAKK, MAPKK, and MAPK in turn, and then send signals to activate specific transcription factors in the nucleus for functional gene expression (Johnson and Lapadat, 2002). LRR-RLK is the largest class of the plant RLKs family and plays critical roles in plant growth and development, hormone signaling, abiotic stresses, and pathogen defense (Shiu and Bleecker, 2001). In *Arabidopsis*, BRI1-associated kinase 1 (BAK1) is a member of the LRR-RLK family that can interact with brassinosteroids insensitive 1 (BRI1). BRI1 and BAK1 are receptors and co-receptors of brassinosteroids (BRs), respectively, and participate in the plant BRs signaling pathway (Li et al., 2002; Nam and Li, 2002). BAK1 binds to the bacterial flagellum protein receptor kinase (FLS2) to form a heterodimer, which can induce disease resistance response in plants (Chinchilla et al., 2007). The rice *Xa21* gene encodes LRR-RLK, the LRR motif in the extracellular region recognizes and binds the toxic compounds produced by the pathogen, thereby enhancing the resistance to *Xanthomonas oryzae* pv. *oryzae* in rice (Song et al., 1995). In this study, the expression level of *LRR-RLK* gene was unchanged in the susceptible variety ROC22 but was gradually increased in the resistant variety YT93-159 by about 6.01-fold of the control at 5 d post *S. scitamineum*-inoculation (**Figure 8A**), suggesting that *LRR-RLK* play a role in sugarcane and *S. scitamineum* interaction.

It is interesting that, when Asai et al. (2002) treated *Arabidopsis* protoplasts with FLS2, the expression of *MEKK1*, *MKK4/MKK5*, *MPK3/MPK6*, *WRKY22*, and *WRKY29* was sequentially activated, and the activation events ultimately induced the expression of defense genes. This is the first complete MAPK signaling module identified in plants to fight against pathogen attacks. Wan et al. (2004) found that chitin elicitors induced *AtMAPK* expression and increased enzymatic activity of AtMPK3 and AtMPK6 with increased levels of *WRKY22/29/33/53* transcripts. A rice D-subclass *MAPK* gene, *BWMK1*, was induced to express at 4 h after infection upon *Magnaporthe grisea*. Overexpressed *BWMK1* induced constitutive expression of pathogenesis-related (PR) genes and enhanced resistance to rice blast disease (He et al., 1999). The rice *MPKK10.2* gene acted in the cross-point of two MAPK cascades leading to X. *oryzae* pv. *oryzicola* resistance and drought tolerance (Ma et al., 2017). A cotton Raf-like *MAP3K* gene, *GhMAP3K65*, enhanced the sensitivity to pathogen infection and heat stress by negatively modulating growth and development in transgenic *Nicotiana benthamiana* (Zhai et al., 2017). Ali et al. (2021) identified 15 *ShMAPKs*, 6 *ShMAPKKs*, and 16 *ShMAPKKKs* genes from the genome of cultivar R570, of which *ShMAPK07* and *ShMAPKKK02* were defense-responsive genes when sugarcane plants were challenged by both *Acidovorax avenae* subsp. *Avenae* and *Xanthomonas albilineans*. In this study, the expression of *MAPK*, *MEK*, and *Raf-like* genes were up-regulated upon *S. scitamineum* infection (**Figures 8B–D** **)**. In particular, the expression level of *MEK* gene was gradually increased to about 3.8-fold of the control at the peak (5 d) in YT93-159, while a slightly up-regulated expression was observed in ROC22 (**Figure 8C**). The results indicated that these three genes may synergistically activate the MAPK cascade signaling pathway to enhance sugarcane’s resistance to smut.

ROS, including superoxide anion (O_2_^-^), singlet oxygen (^1^O_2_), hydrogen peroxide (H_2_O_2_), and hydroxyl radical (-OH) is produced in plants in response to abiotic stresses and various bacterial and fungal diseases (Choudhury et al., 2017). ROS acts as a signaling molecule in plant growth and development. Too low a level of ROS inhibits cell growth, while too high a level of ROS is cytotoxic. It is essential to keep a reasonable level of intracellular ROS and maintain a dynamic balance between ROS production and cleavage (Wang et al., 2016). Main enzymes that play a role in ROS scavenging in plants include superoxide dismutases (SODs), ascorbate-glutathione, glutathione peroxidases (GPXs), and CATs (Mittler, 2002). CATs are a class of highly active enzymes essential for ROS detoxification (Mhamdi et al., 2010). *CAT2* overexpression led to a higher CAT enzyme activity and enhanced resistance to oxidative stress and pathogen infection in transgenic *N. benthamiana* plants (Polidoros et al., 2001). Two sugarcane catalase genes, *ScCAT1* and *ScCAT2*, played a positive role in immune responses in sugarcane-*S. scitamineum* interactions, as well as in various abiotic stresses (Su et al., 2014; Sun et al., 2018). In this study, the expression of *CAT1* was also up-regulated and reached the peak of 13.06-fold increase at 1 d post *S. scitamineum*-inoculation in YT93-159 (**Figure 8E**). POD is another oxidoreductase that scavenges ROS in plants and plays an important role in plant response to hormones, drought, oxidative stress, and pathogen attack (Valério et al., 2004). Hu et al. (2015a); Hu et al. (2015b) identified four III-class peroxidases gene (*KJ001797*, *KJ001798*, *SsPOD-1*, and *KJ001799*) from *S. officinarum*, *S. spontaneum*, and *S. arundinaceum*, respectively, with highly conserved functional regions. Su et al. (2017) identified the *ScPOD02* gene from a smut-resistant genotype Yacheng 05-179 two days post *S. scitamineum*-inoculation. The transcripts of *ScPOD02* were up-regulated in smut-resistant varieties but remained unchanged or slightly reduced in susceptible varieties. In this study, *POD* gene expression was up-regulated upon *S. scitamineum* infection. The expression level of *POD* was slightly higher in YT93-159 than ROC22 (**Figure 8F**). Thus, it is speculated that both *CAT1* and *POD* genes are involved in the ROS signaling pathway by scavenging excess ROS to effectively improve the resistance to sugarcane smut.

Ca^2+^ plays an important role as a second messenger when plants are subjected to abiotic or pathogen attack (Boudsocq and Sheen, 2013). Ca^2+^-sensor proteins include calmodulin (CAM), calcineurin B-like protein (CBL), CML, and CDPK. Among them, CML is a family of plant-specific Ca^2+^ sensor proteins that are widely involved in various processes of plant growth and development (Boudsocq and Sheen, 2013). Heyer et al. (2022) reported that Ca^2+^ sensor proteins CML37 and CML42 antagonistically regulated plants’ defense against insect infestation by *Spodoptera littoralis* and drought. Vadassery et al. (2012) found that Ca^2+^ and phytohormone were induced along with *CML42* gene expression when stimulated by *S. littoralis* in *A. thaliana*. *CML42* acted as a negative regulator in plant defense by decreasing *COI1*-mediated JA sensitivity and JA-responsive gene expression. In the present study, the expression of *CML42* was up-regulated upon *S. scitamineum* infection. The expression level of *CML42* was gradually increased in YT93-159 to about 3.82-fold of the control at 5 d but remained unchanged in ROC22 (**Figure 8I**). CBL and CIPK form an important signaling regulatory network in response to abiotic stress (Mao et al., 2016). The interaction between CIPK24/SOS2 (salt-overly-sensitive) and CBL4/SOS3 may directly regulate the downstream component SOS1, a putative Na^+^/H^+^ antiporter, thereby enhancing the salt detoxification process in *Arabidopsis* (Shi et al., 2000; Chinnusamy et al., 2004). Su et al. (2020) identified 48 *SsCIPKs* from *S. spontaneum* and cloned 10 *ScCIPK* genes from the sugarcane cultivar ROC22. Six *ScCIPK* genes (*1*, *2*, *15*, *20*, *21*, and *28*) were up-regulated under polyethylene glycol (PEG) stress. Three *ScCIPK* genes (*1*, *2*, and *28*) were up-regulated upon NaCl stress. Transient overexpression of *ScCIPKs* in *N. benthamiana* plants demonstrated that the *ScCIPK* genes responded to various abiotic stresses and bacterial infections by participating in ethylene synthesis pathway. In this study, *CIPK* gene was induced to express at a similar level in different samples upon *S. scitamineum* infection (**Figure 8J**). It is thus suggested that Ca^2+^ sensor proteins CML42 and CIPK are involved in the Ca^2+^ signaling pathway and enhance the resistance to *S. scitamineum* in sugarcane.

**Figure 9:**
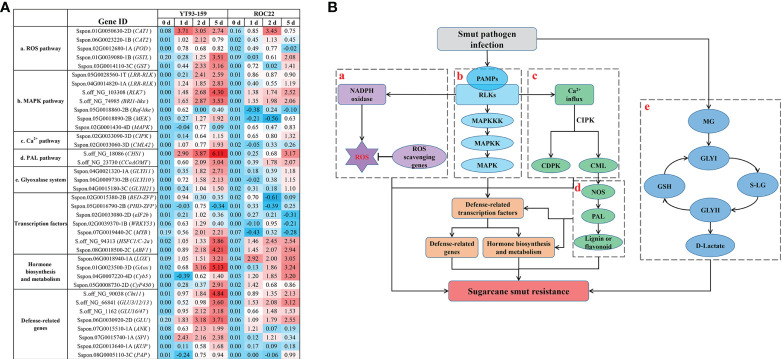
** **A potential molecular mechanism of sugarcane and *S. scitamineum* interaction. **(A)** A heat map of the key genes identified by BSR-Seq and WGCNA. Values indicate log ratios of relative expression levels of the key genes by RT-qPCR in YT93-159 and ROC22 at 0 d, 1 d, 2 d, and 5 d post *S. scitamineum*-inoculation. **(B)** The potential molecular mechanism diagram. a, ROS signaling pathway; b, MAPK cascade signaling pathway; c, Ca^2+^ signaling pathway; d, PAL metabolic pathway; e, the glyoxalase system; PAMPs, pathogen-associated molecular proteins; RLKs, receptor-like kinases; ROS, reactive oxygen species; MAPK, mitogen-activated protein kinase; CIPK, calcineurin B-Like interacting protein kinase; CDPK, calcium-dependent protein kinase; CML, calmodulin like; NOS, nitric-oxide synthesis; PAL, phenylpropanoid; MG, methylglyoxal; GSH, glutathione; GLYI, glyoxalase I; GLYII, glyoxalase II; SD-lactoylglutathione (S-LG).

A potential molecular mechanism of sugarcane and *S. scitamineum* interaction was depicted by combining the results of BSR-Seq with WGCNA data (Wu et al., 2022) (**Figure 9**). When sugarcane is infected with *S. scitamineum*, pathogen-associated molecular proteins (PAMPs) bind to receptor-like proteins (RLKs) to activate MAPKKK, MAPKK, and MAPK in sequence (Johnson and Lapadat, 2002). MAPK is activated and enters the nucleus, and thus promotes gene expression by activating transcription factors *ZFP*, *MYB* and *WRKY53*. The transcription factors regulate abscisic acid (ABA), salicylic acid (SA), jasmonic acid (JA), gibberellin (GA), ethephon (ET), and other hormone metabolic pathways, thereby enhancing the resistance to sugarcane smut disease. Upon sugarcane and *S. scitamineum* interaction, reactive oxygen species (ROS) are generated, which cause a hypersensitive response (HR) to a certain extent, thereby inducing plant resistance. However, when ROS accumulates in excess, they become toxic and cause damage to plant growth. Protein kinases such as CAT, POD, and glutathione S-transferases (GST) are effective in scavenging excess ROS to maintain a dynamic balance of ROS in plants. Moreover, the binding of PAMPs to RLKs also causes a [Ca^2+^] burst that activates the production of calcium sensor proteins, such as CML and calcium-dependent protein kinase (CDPK), which in turn activate the nitric-oxide synthesis (NOS) and phenylpropanoid (PAL) metabolic pathway to produce more lignin and flavonoids, thereby enhancing the resistance to sugarcane smut. Furthermore, under the stress of smut disease, a large amount of toxic methylglyoxal (MG) is produced in sugarcane, which activates the glyoxalase system to remove MG to alleviate or relieve the effect of excess MG on sugarcane growth and development. In summary, the activation of MAPK cascade signaling, ROS signaling, Ca^2+^ signaling, and PAL metabolic pathway and the initiation of the glyoxalase system jointly promote the resistance to sugarcane smut disease.

## Conclusion

In this study, a field disease survey was conducted on 312 F_1_ progenies of a crossing between smut-resistant variety YT93-159 and smut-susceptible variety ROC22. Based on the disease data, one smut-resistant bulk (27 progenies) and one smut-susceptible bulk (24 progenies) were constructed. BSR-Seq technology was then used to sequence YT93-159, ROC22, and the two bulks to yield 164.44 GB clean data. A total of 17,477 genes were optimized, 12,138 new genes were annotated, and 7,295 DEGs were identified using the STAR (v2.3.0e) software and a *S. spontaneum* reference genome. GO and KEGG enrichment analyses revealed that the DEGs were mainly enriched in stress-related metabolic pathways (carbon metabolism, phenylalanine metabolism, plant hormone signal transduction, and glutathione metabolism) and plant-pathogen interactions. In addition, 45,946 high quality SNPs were identified. A 1.27 Mb chromosome region associated smut resistance was localized to *S. spontaneum* Chr5B (68,904,827 to 70,172,982). One hundred and twenty-nine candidate genes were identified based on both ΔSNP-index and ED methods. Furthermore, 24 key genes encoding enzymes in signaling pathways or transcription factors were found, which were closely related to stress resistance. RT-qPCR analysis confirmed that 20 key genes were induced to express upon *S. scitamineum* infection, and the expression levels were significantly higher in YT93-159 than ROC22. Combining the results of BSR-Seq with our previous WGCNA study (Wu et al., 2022), a potential molecular mechanism of sugarcane and *S. scitamineum* interaction is drawn in **Figure 9**, which indicates that the activation of MAPK cascade signaling, ROS signaling, Ca^2+^ signaling, and PAL metabolic pathway and the initiation of the glyoxalase system may jointly promote the resistance to *S. scitamineum* in sugarcane. The results should benefit further understanding of the molecular mechanisms of smut resistance and provide many SNPs and gene resources for future smut resistance breeding in sugarcane.

## Data availability statement

The raw sequencing data were deposited in National Genomics Data Center (NGDC), Beijing Institute of Genomics, Chinese Academy of Sciences, under Project PRJCA012242 with Genome Sequence Archive (GSA) number CRA008356 (https://bigd.big.ac.cn/gsa/browse/CRA008356).

## Author contributions

QW, Y-BP, GPM, LX and YQ conceived and designed the experiments, QW, YS, WZ, PL, and TS performed the experiments, QW, FX, BQ and YS analyzed the data, QW wrote the original manuscript, Y-BP, GPM, YS, LX and YQ revised the manuscript. All authors contributed to the article and approved the submitted version.

## Funding

This research was funded by the National Key R&D Program of China (2019YFD1000500), National Natural Science Foundation of China (31971992 and 31781688), Natural Science Foundation of Fujian Province, China (2020J01591 and 2015J06006), China Agriculture Research System of MOF and MARA (CARS-17), and a Non-Funded Cooperative Agreement between the USDA-ARS and NRDCSIT on Sugarcane Breeding, Varietal Development, and Disease Diagnosis, China (Accession Number: 428234).

## Acknowledgments

The authors are thankful to Zhongqi He and Perng-Kuang Chang for reviewing the manuscript with excellent comments. USDA is an equal opportunity provider and employer. And, we are grateful to the reviewers for their helpful comments on the original manuscript. We would like to thank editors for their efficient works.

## Conflict of interest

The authors declare that the research was conducted in the absence of any commercial or financial relationships that could be construed as a potential conflict of interest.

## Publisher’s note

All claims expressed in this article are solely those of the authors and do not necessarily represent those of their affiliated organizations, or those of the publisher, the editors and the reviewers. Any product that may be evaluated in this article, or claim that may be made by its manufacturer, is not guaranteed or endorsed by the publisher.
