# Supplementary material for: Genetic identification of SNP markers and candidate genes associated with sugarcane smut resistance using BSR-Seq
Source: Front Plant Sci. 2022 Oct 13;13:1035266. doi: 10.3389/fpls.2022.1035266 (PMC9608552; doi:10.3389/fpls.2022.1035266)
Supplement: Supplementary file 1 [file DataSheet_1.pdf]

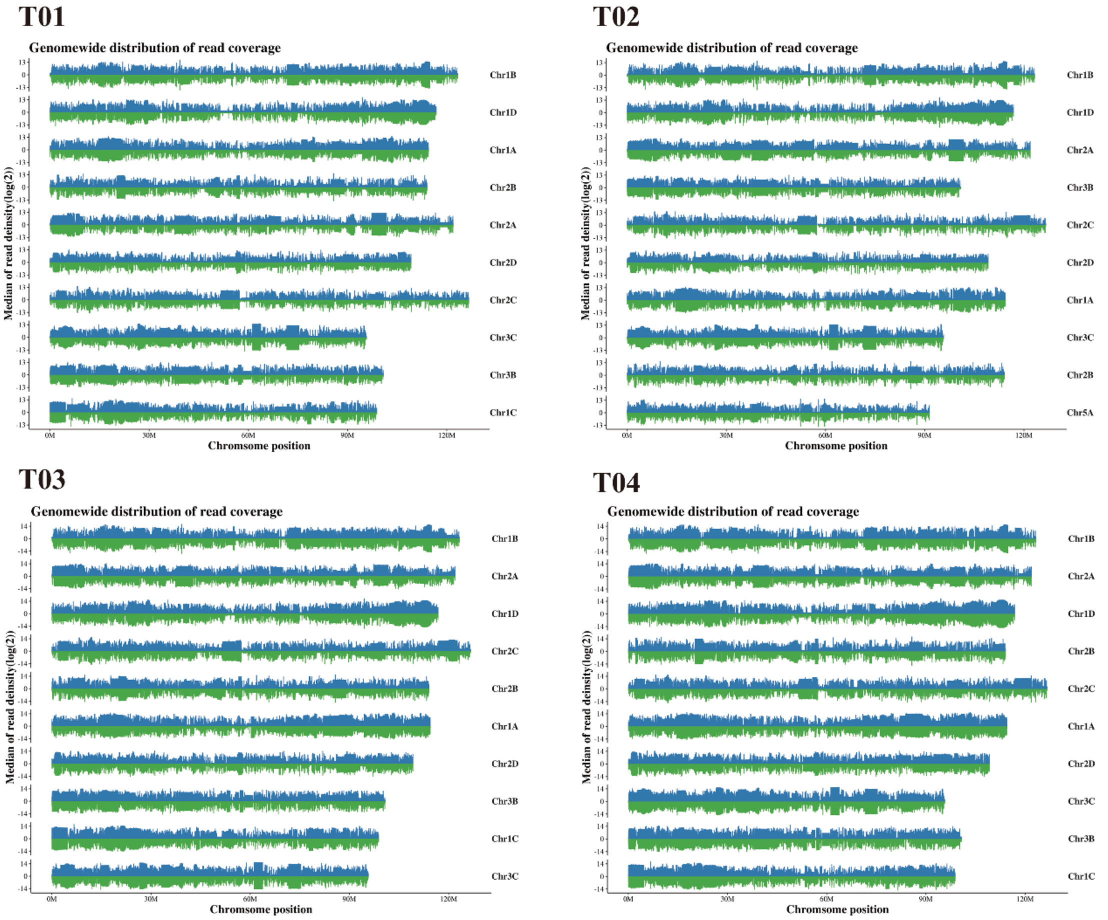

FIGURE S1 The position and coverage depth of mapped reads on the *Saccharum spontaneum* genome. Blue is a positive chain, and green is a negative chain. T01, YT93-159; T02, ROC22; T03, the resistant bulk; T04, the susceptible bulk.
